# Metabolomics and cardiovascular risk factors in autoimmune-mediated connective tissue diseases – an exploratory, hypothesis-generating study

**DOI:** 10.1186/s41927-026-00670-8

**Published:** 2026-07-01

**Authors:** Emily Deichsler, Steffen Heelemann, Selina Strathmeyer, Meike Hoffmeister, Werner Dammermann, Oliver Ritter, Daniel Patschan, Susann Patschan

**Affiliations:** 1 Department of Internal Medicine 1 – Cardiology, Angiology, Nephrology, Pneumology, Brandenburg Medical School (Theodor Fontane), Brandenburg University Hospital, Brandenburg, Germany; 2 Lifespin GmbH, Regensburg, Germany; 3 Institute of Biochemistry, Brandenburg Medical School (Theodor Fontane), Brandenburg, Germany; 4 Faculty of Health Sciences (FGW), Joint faculty of the University of Potsdam, the Brandenburg Medical School Theodor Fontane and the Brandenburg Technical University Cottbus-Senftenberg, Cottbus, Germany; 5 Department of Medicine 2, Gastroenterology, Diabetes, Endocrinology, University Hospital Brandenburg of the Brandenburg Medical School Theodor Fontane, Brandenburg, Germany

**Keywords:** Metabolomics, Systemic lupus erythematosus, Systemic sclerosis, Sjögren´s disease, Myositis

## Abstract

**Background:**

Autoimmune connective tissue diseases (ACTD), including systemic lupus erythematosus (SLE), systemic sclerosis (SSc), Sjögren’s disease (SD), and idiopathic inflammatory myopathies (IIM), are associated with markedly increased cardiovascular risk (CVR) that is insufficiently captured by conventional risk scores. Reliable biomarkers for CVR stratification in ACTD are lacking. This exploratory, hypothesis-generatig study aimed to investigate whether metabolomic alterations reflect CVR across ACTD subtypes.

**Methods:**

In this cross-sectional, exploratory study, patients with SLE (*n* = 33), SSc (*n* = 18), SD (*n* = 16), and IIM (*n* = 9) were recruited from a tertiary rheumatology center. Serum metabolomic profiling was performed using ¹H-NMR spectroscopy. Associations between 113 quantified metabolites and clinical CVR parameters (including age, sex, body mass index, glucocorticoid use, Framingham score, hypertension, diabetes, lipid parameters, and lifestyle factors) were analyzed using non-parametric statistics with false discovery rate correction. Correlation analyses were conducted using Spearman coefficients.

**Results:**

Metabolomic alterations varied substantially across ACTD subtypes and CVR factors. Lipid metabolites showed the strongest and most consistent associations with CVR parameters. The Framingham score correlated with 11 metabolites in SLE, 1 in SSc, 2 in SD, and 93 in IIM, predominantly involving lipid components. Diabetes mellitus was associated with extensive metabolomic changes, particularly in SSc (*n* = 62 metabolites), followed by SD (*n* = 20) and SLE (*n* = 5). In contrast, arterial hypertension showed minimal metabolomic differentiation across most ACTD, except in IIM. Body mass index correlated mainly with lipid metabolites in SSc, but not in SLE. Glucocorticoid therapy and dosage were strongly associated with alterations in lipid metabolism, especially in SLE and SSc. Across all entities, correlations with total cholesterol, LDL, and HDL were dominated by lipid metabolites. Associations with renal markers (UACR) were limited.

**Conclusions:**

ACTD are characterized by heterogeneous metabolomic signatures associated with cardiovascular risk factors. Larger, longitudinal studies are required to validate these findings and to determine their clinical utility in cardiovascular risk stratification in ACTD.

**Supplementary Information:**

The online version contains supplementary material available at 10.1186/s41927-026-00670-8.

## Introduction

Autoimmune-mediated connective tissue diseases (ACTD) represent a heterogeneous group of inflammatory rheumatic disorders. The main entities include systemic lupus erythematosus (SLE), Sjögren’s disease (SD), idiopathic inflammatory myopathies (IIM), systemic sclerosis (SSc), and mixed connective tissue disease (MCTD). The prevalence of all forms of ACTD is estimated to be approximately 0.1% to 0.5% of the population, translating to about 100 to 500 cases per 100,000 individuals. In contrast, the prevalence of rheumatoid arthritis (RA) and seronegative spondyloarthritis (SpA) is approximately 1% for each condition [1, 2].

Despite their significant differences in etiology, pathogenesis, and especially clinical presentation, all forms of ACTD are characterized by a markedly increased cardiovascular risk (CVR). This association has also been established for RA and SpA. It is no coincidence that over a decade ago, a European Alliance of Associations for Rheumatology (EULAR) guideline on cardiovascular risk management in inflammatory joint disorders was published [3]. The increase in cardiovascular risk is of multifactorial origin. On one hand, affected patients accumulate conventional risk factors at a rate similar to that of the aging general population. On the other hand, systemic glucocorticoids are commonly used during various phases of nearly all conditions. Their pro-atherogenic potential is well established. Additionally, non-steroidal anti-inflammatory drugs (NSAIDs) also exhibit pro-atherogenic effects, with no significant differences observed between Cyclooxygenase-2 (COX-2) selective and non-selective agents. Ultimately, the inflammatory activity of the underlying diseases should be recognized as a cardiovascular risk factor.

The conventional cardiovascular risk assessment is regulated in guidelines, such as those from the European Society of Cardiology (ESC) [4]. In individuals with inflammatory rheumatic diseases, established risk scores such as SCORE2 [5], Framingham [6], and PROCAM [7] tend to underestimate the risk faced by these patients [8]. The ESC and EULAR recommend adjusting the results of these scores by applying a multiplication factor of 1.5 in cases such as RA, provided certain criteria are met (e.g., disease duration > 10 years, positive rheumatoid factor / anti-cyclic citrullinated peptide antibodies (RF / ACPA), extra-articular manifestations) [9]. However, there are currently no serological surrogate markers available that would allow cardiovascular risk assessment in ACTD patients. Conventional or established indicators such as LDL cholesterol, HbA1c, or NT-proBNP do not adequately capture the specific risks associated with inflammatory autoimmune conditions. Identifying such indicators would undoubtedly enhance the quality of medical care for those affected.

Metabolomics enables the detection and quantification of small-molecule substances (< 1.5 kD) from biological materials such as blood, urine, and tissue samples [10]. The analytes include defined carbohydrates, amino acids, fatty acids, and their metabolites. The ‘omics’ concept aims to make the complexity of biological processes more comprehensible, although the extensive datasets can sometimes be challenging to interpret. Our group has recently published two studies on metabolic profiling in RA [11] and SpA [12].

ACTD exhibit considerable heterogeneity regarding their etiology and pathogenesis, as well as marked clinical differences. However, the elevation in cardiovascular risk appears to be largely comparable among SLE, SSc, and IIM. This study therefore seeks to evaluate whether the observed increase in cardiovascular risk - indicated by the prevalence of specific cardiovascular risk factors - is reflected by distinct metabolomic alterations across ACTD subtypes. The study does not yet aim to analyze metabolomic abnormalities associated with varying degrees of cardiovascular end-organ damage or related or concomitant conditions. Rather, the goal is to explore candidate molecules or molecular patterns that could serve as a starting point for longitudinal studies.

The present study does not focus on disease activity variables or the immunological characteristics of the entities. Those analyses are beyond the scope of this article and will be reported in a separate publication currently in preparation.

## Methods

### Design

This cross-sectional, exploratory, hypothesis-generating study was conducted at the Healthcare Center of Brandenburg University Hospital, part of Brandenburg Medical School Theodor Fontane, from November 2022 through January 2023. The corresponding blood samples were taken from 2019 onwards whenever a patient presented with the relevant diagnosis and agreed in writing to participate in the study. The above-mentioned period (November 2022 to January 2023) covers the period of metabolomic analyses and clinical data collection. The study was approved by the Brandenburg Medical School Ethics Committee (E-01-20190911). All participants provided written informed consent by signing a consent form. The study was performed in accordance with the in accordance with the Declaration of Helsinki. 

### Patients

All study participants were recruited from the rheumatology outpatient clinic at the Healthcare Center of Brandenburg University Hospital (Brandenburg Medical School Theodor Fontane). The following inclusion criteria were used: a diagnosis of SLE according to the ´2019 European League Against Rheumatism/American College of Rheumatology classification criteria for systemic lupus erythematosus´ [13], or a diagnosis of SSc according to the ´2013 Classification Criteria for Systemic Sclerosis: An American College of Rheumatology/European League Against Rheumatism Collaborative Initiative´ [14], or a diagnosis of SD according to the 2012 published ´American College of Rheumatology classification criteria for Sjögren’s syndrome´ [15], or a diagnosis of IIM according to the ´2017 European League Against Rheumatism/American College of Rheumatology classification criteria for adult and juvenile idiopathic inflammatory myopathies and their major subgroups´ [16]. To be eligible, patients had to be at least 18 years old. Patients were excluded if they had uncontrolled psychiatric disorders, additional autoimmune diseases, uncontrolled infectious diseases such as HIV, hepatitis B or C, and tuberculosis, uncontrolled drug or alcohol dependence, or pregnancy. The following patient characteristics were collected: nationality, height, weight, comorbidities, medications, smoking status, and family history of ACTD. Treatment of the diseases included prednisolone in variable, individualized doses, NSAIDs as needed (different preparations and doses, either as needed or taken regularly), and one or more disease-modifying antirheumatic drugs (DMARDs), adapted to the stage and activity of the disease. The DMARDs were either conventional preparations (cDMARDs) or biologic preparations (bDMARDs) or corresponding biosimilars (bsDMARDs). The cardiovascular risk assessment included the following morbidities and laboratory parameters: age (years), gender, arterial body mass index (kg/qm), glucocorticoid therapy (no/yes), daily glucocorticoid dose (mg), smoking (no/yes), stress (no/yes), arterial hypertension (no/yes), diabetes mellitus (no/yes), total cholesterol (mmol/l), LDL (mmol/l), HDL (mmol/l), urine albumin-creatinine ratio (UACR in g/g), and Framingham score. The Framingham score has limitations. More recent guidelines recommend using SCORE2 [17] instead. However, for individuals over 70 years of age, SCORE2-OP should be used instead. Both the Framingham score and SCORE2/SCORE2-OP only capture the atherogenic effects of underlying inflammatory diseases to a limited extent. For the Framingham score, all values must be multiplied by 1.5 to account for systemic inflammation. However, since we have not conducted comparisons with healthy individuals, we did not apply this multiplication. Doing so would not change the correlations.

### Sample preparation and NMR measurement

Following a thawing period of approximately three hours at room temperature, 350 µl of serum was extracted and combined with 350 µl of aqueous buffer. The buffer consists of H_2_O p.A., 0.1 g/l NaN_3_, 0.067 mol/l Na_2_HPO_4_, 0.033 mol/l NaH_2_PO_4_ (pH: 7.15 ± 0.05), 5% D_2_O as a field-lock substance and an internal standard (6 mM pyrazine) for quantification. From this mixture, 600 µl were transferred into a 5 mm Bruker NMR tube and sealed with barcode-labeled lids. Subsequent nuclear magnetic resonance (NMR) measurements were performed using the following parameters: a Bruker AVANCE NEO 600 MHz spectrometer, with a 1D 1 H noesypr1d_d20 measuring method, an NS of 16, a T of 310 K, and a measuring time per sample of 6.5 min. All measured spectra successfully passed the quality control (QC) routine and were subsequently released for data analysis.

### Data analysis

The spectra obtained were subsequently transformed using Fourier analysis with the TopSpin software (version 4.0, Bruker Biospin, Germany). All spectra were automatically phased and subjected to baseline correction. Subsequently, the spectra were analyzed using the proprietary Lifespin Profiler software (version 1.4_Blood), and a quantitative metabolite list was generated. All metabolites are quantified in mmol/L.

### Statistical analysis

Statistical significance in this analysis was determined using the Wilcoxon-Mann-Whitney test. The resulting p-values were corrected for multiple testing using the false discovery rate (FDR) correction method, which was applied to all 113 metabolites that were tested. The corrected p-values were converted into ∗ notation as follows: the symbols ∗∗∗, ∗∗, and ∗ indicate *p* ≤ 0.001, *p* ≤ 0.01, and *p* ≤ 0.05, respectively. Cohen’s d quantifies the standardized effect size by expressing the difference between two group means in units of pooled standard deviation, whereas fold change expresses the relative change between two measurements as a simple ratio; together they illustrate both the magnitude of change and how large that change is relative to variability in the data. The Spearman correlation coefficient itself also represents an effect size. The coefficient ρ (rho) itself serves as a measure of effect strength, it quantifies how strongly two variables are monotonically related - the closer |ρ| is to 1: |ρ| ≥ 0.10 = small effect, |ρ| ≥ 0.30 = medium effect, |ρ| ≥ 0.50 = large effect. Positive correlations were defined as correlation coefficients greater than 0.6, and negative correlations as coefficients less than − 0.6. With the aim of presenting the results in a more accessible way, 4 groups of metabolites have been defined: amino acids, lipid components, carbohydrates as well as others. The supplementary table summarizes all differences or correlations. In principle, no multivariate analyses were performed, as the size of the recruited cohorts did not appear to be sufficient.

## Results

The cohort sizes were as follows: SLE *n* = 33 (30 women, 3 men), SSc *n* = 18 (15 women, 3 men), SD *n* = 16 (15 women, 1 man), and IIM *n* = 9 (5 women, 4 men). The mean age of patients was 55.4 ± 13.4 years for SLE, 59.4 ± 14.3 years for SSc, 63.3 ± 21 years for SD, and 59.7 ± 12.6 years for IIM. Table 1 lists all essential clinical characteristics at the time of study inclusion.

**Table 1:** Baseline characteristics of all included patients

Variable	SLE	SSc	SD	IIM
**general characteristics**				
gender (females in %)	90.9	81	90	50
age (years)	55.4 ± 13.4	59.4 ± 14.3	63.3 ± 21	59.7 ± 12.6
BMI (kg/qm)	26.3 ± 4.2	27.4 ± 5.3	25.5 ± 5.4	26.2 ± 5
DOD (years)	10 ± 1.6	4.6 ± 5.2	7.4 ± 5.9	0.9 ± 1.1
**morbidities / additional risk factors (%)**				
arterial hypertension	45.5	71.4	60	60
diabetes mellitus	6.1	19	10	0
obesity	24.2	19	25	20
smoking	39.4	23.8	20	40
stress	37.5	38.1	52.6	50
prevalence of glucocorticoid therapy (%)	81	66	75	55
Framingham score	13.1 ± 6.1	14.2 ± 5.9	10.6 ± 9.3	13.8 ± 3.6
Hannover Functional Questionnaire - HFQ (%)	78.1 ± 21.4	71 ± 20.6	72.2 ± 26.2	78.3 ± 18.7
**disease-related characteristics**				
systemic lupus erythematosus (SLE)				
ANA titre (± SD)	1,793 ± 2,731			
anti-dsDNA (%)	72.7			
SLEDAI (± SD)	5.1 ± 3.8			
SLICC/ACR damage index (± SD)	5.7 ± 3.1			
ACR / EULAR score (± SD)	14 ± 7.2			
anti-Phospholipid-syndrome (%)	15.1			
HCQ therapy (%)	87.8			
HCQ therapy duration (months ± SD)	54.8 ± 50.8			
systemic sclerosis (SSc)				
ANA titre (± SD)		1,934 ± 2,354		
anti-Scl70 (%)		33.3		
anti-Centromer (%)		57.1		
ACR / EULAR score (± SD)		12.7 ± 6.9		
Rodnan skin score (± SD)		1.6 ± 3.3		
Sjögren disease (SD)				
ACR / EULAR score (± SD)			4.7 ± 1.2	
ESSDAI score (± SD)			12.9 ± 9.8	
idiopathic inflammatory myopathy (IIM)				
ANA titre (± SD)				242 ± 94
anti-Jo1 (%)				27.2
anti-PL7 (%)				18.1
anti-PL12 (%)				0
anti-SAE (%)				9.09
anti-SRP (%)				9.09
anti-Ro52 (%)				9.09
inclusion body myositis-functional rating scale (IBM-FRS) (± SD)				32.5 ± 5.5
**laboratory findings**				
CRP (mg/l)	2.5 ± 2.4	3.1 ± 3.8	3.5 ± 4.8	1.2 ± 1
ESR (mm in hour 1)	20.7 ± 17.5	21.6 ± 20.3	25.7 ± 12.4	22.4 ± 17.9
total cholesterol (mmol/l)	5.2 ± 1	5.2 ± 1.1	4.5 ± 1.2	5.4 ± 1
LDL colesterol (mmol/l)	2.9 ± 0.9	3 ± 1	2.5 ± 0.9	3.2 ± 0.9
HDL cholesterol (mmol/l)	1.8 ± 0.6	1.7 ± 0.4	1.7 ± 0.4	1.7 ± 0.4
UACR (mg/g)	37.5 ± 72.8	22.1 ± 27.4	38.8 ± 43.7	18.9 ± 7.5

Data are shown as percentage or as mean ± standard deviation (abbreviations: ACR - American College of Rheumatology; ANA - anti-nuclear antibody; anti-dsDNA - antibodies against native double-stranded DNA; BMI – body mass index; DOD – duration of disease; CRP - reactive protein; ESR – erythrocyte sedimentation rate; ESSDAI - EULAR Sjögren’s Syndrome Disease Activity Index; EULAR - European League Against Rheumatism; SLEDAI - Systemic Lupus Erythematosus Disease Activity Index; SLICC - Systemic Lupus International Collaborating Clinics; UACR – Urine Albumin-to-Creatinine Ratio; *additional information: semiquantitative evaluation ANA titre by indirect immunofluorescence using HEp-2 cells*,* indication of the titre according to the decision of doctors for laboratory medicine; analysis of the anti-dsDNA titres by ELISA*,* results as IE/ml – currently only rating as positive in %; analysis of all other autoantibody entities using ELISA*,* results assessed as positive in %)*

### Cardiovascular risk factor analysis

Our analyses took into account conventional cardiovascular risk factors, such as hypertension, age, diabetes mellitus, and gender. However, studies on other potent risk factors, some of which are more strongly associated with cardiovascular disease, were not included. These factors include hyperhomocysteinemia and hyperlipoproteinemia (a).

### General characteristics and medication

#### Age

Patients with (SLE) exhibited 16 metabolites that were positively correlated with age, all substrates were metabolites of lipid metabolism. In individuals with SSc, three metabolites were identified that showed a positive correlation with age (2 amino acids, 1 other). For SD, only one metabolite showed a positive correlation with age (other). In contrast, patients with IIM exhibited four negatively correlated substrates and thirteen positively correlated substrates (3 amino acids, 11 lipids, 3 other).

#### Gender

The following metabolites differed significantly between women and men: SLE − 5 metabolites (three lower, two higher in women – 2 amino acids, 3 other); SSc − 38 metabolites (eight lower, 30 higher in women; 6 amino acids, 27 lipids, 5 other); SD − 0 metabolites; and IIM − 2 metabolites (both higher in women, 1 amino acid, 1 other) (supplemental Table 1).

#### Body mass index

In individuals with SLE, there were no significant positive or negative correlations between BMI and any measured serum metabolites. In SSc patients, BMI showed strong positive correlations with 16 metabolites (2 amino acids, 13 lipids, 2 carbohydrates, 1 other). The SD group was characterized by strong positive correlations between BMI and 5 metabolites (2 amino acids, 1 lipid, 1 carbohydrate, 1 other). In IIM patients, five metabolite–BMI correlations were observed: two were strongly negative and three were strongly positive (2 amino acids, 3 other) (supplemental Table 1).

#### Glucocorticoid therapy

Among SLE patients, those who regularly received glucocorticoids (GCs) (27 of 33) exhibited significantly higher concentrations of 11 metabolites (7 lipids, 4 other). Twelve of 18 patients with systemic sclerosis (SSc) were receiving regular glucocorticoid therapy; in these patients, eight metabolites were present at higher concentrations than in patients not receiving regular GCs (7 lipids, 1 other). In the Sjögren’s disease cohort, 12 of 16 patients underwent GC treatment; six metabolites were significantly different in this group than in GC-naive patients. Three metabolites were at higher and three at lower concentrations (1 amino acid, 3 lipids, 2 other). In the IIM group, five of nine patients were receiving regular GC therapy. Three metabolites showed altered concentrations: two were elevated and one was reduced (1 amino acid, 1 lipid, 1 other) (supplemental Table 1).

#### Daily glucocorticoid dose

In the following analyses, the daily prednisolone dose (mg) was correlated with the concentrations of distinct metabolites. From this point forward, we report, for each entity, the number of metabolites that showed a significant correlation. SLE − 22 (21 negatively, 1 positively correlated; 22 lipids), SSc − 26 (25 negatively, 1 positively correlated; 1 amino acid, 23 lipids, 2 other), SD – 1 (1 other), IIM − 0 (supplemental Table 1).

#### Framingham score

Correlation analyses between the Framingham score and metabolite concentrations revealed the following numbers of metabolites with significant positive or negative correlations: SLE − 11 (all positively correlated), SSc − 1 (positively correlated), SD − 2 (positively correlated), IIM − 93 (87 positively correlated, 6 negatively correlated) (Table 2). The additive analysis of the 4 metabolite groups resulted in the following numbers of cumulative differences or correlations for the respective diseases: SLE – 11 lipids, 0 amino acids / carbohydrates / other; SSc – 1 other, 0 amino acids / lipids / carhohydrates; SD – 2 other, 0 amino acids / lipids / carhohydrates; IIM – 4 amino acids, 86 lipids, 0 carbohydrates, 3 other (supplemental Table 2).

**Table 2:** Correlations between the metabolomic profile and the Framingham score in the respective entities

Metabolite	Spearman corrcoeff (Zeros Incl.)
**SLE**	
Total_Chols [mg/dL]	0.672
Total_CE [mg/dL]	0.679
Total_FC [mg/dL]	0.635
Total_PL [mg/dL]	0.657
P_Num_IDL [nmol/L]	0.652
Chols_IDL [mg/dL]	0.644
CE_IDL [mg/dL]	0.644
FC_IDL [mg/dL]	0.648
PL_IDL [mg/dL]	0.64
TG_IDL [mg/dL]	0.632
ApoB_IDL [mg/dL]	0.644
**SSc**	
Cystine	0.818
**SD**	
Pyruvic acid	0.636
Taurine	0.667
**IIM**	
Albumin	-0.753
Citric acid	-0.77
Glutamine	-0.603
Glycerol	0.778
Histidine	-0.653
Phenylalanine	0.644
Proline	-0.778
Sphingomyeline	0.82
Total_Chols [mg/dL]	0.862
Total_CE [mg/dL]	0.862
Total_FC [mg/dL]	0.895
Total_ApoB [mg/dL]	0.803
non_HDL_P_Num [nmol/L]	0.803
P_Num_LDL6 [nmol/L]	0.828
Chols_LDL6 [mg/dL]	0.795
CE_LDL6 [mg/dL]	0.795
FC_LDL6 [mg/dL]	0.795
PL_LDL6 [mg/dL]	0.795
TG_LDL6 [mg/dL]	0.795
ApoB_LDL6 [mg/dL]	0.828
P_Num_LDL5 [nmol/L]	0.77
Chols_LDL5 [mg/dL]	0.77
CE_LDL5 [mg/dL]	0.77
FC_LDL5 [mg/dL]	0.77
PL_LDL5 [mg/dL]	0.77
TG_LDL5 [mg/dL]	0.77
ApoB_LDL5 [mg/dL]	0.77
P_Num_LDL4 [nmol/L]	0.803
Chols_LDL4 [mg/dL]	0.803
CE_LDL4 [mg/dL]	0.803
FC_LDL4 [mg/dL]	0.803
PL_LDL4 [mg/dL]	0.803
TG_LDL4 [mg/dL]	0.803
ApoB_LDL4 [mg/dL]	0.803
P_Num_LDL3 [nmol/L]	0.803
Chols_LDL3 [mg/dL]	0.803
CE_LDL3 [mg/dL]	0.803
FC_LDL3 [mg/dL]	0.803
PL_LDL3 [mg/dL]	0.803
TG_LDL3 [mg/dL]	0.803
ApoB_LDL3 [mg/dL]	0.803
P_Num_LDL2 [nmol/L]	0.803
Chols_LDL2 [mg/dL]	0.803
CE_LDL2 [mg/dL]	0.803
FC_LDL2 [mg/dL]	0.803
PL_LDL2 [mg/dL]	0.803
TG_LDL2 [mg/dL]	0.803
ApoB_LDL2 [mg/dL]	0.803
P_Num_LDL1 [nmol/L]	0.895
Chols_LDL1 [mg/dL]	0.895
CE_LDL1 [mg/dL]	0.895
FC_LDL1 [mg/dL]	0.895
PL_LDL1 [mg/dL]	0.895
TG_LDL1 [mg/dL]	0.895
ApoB_LDL1 [mg/dL]	0.895
P_Num_LDL [nmol/L]	0.828
Chols_LDL [mg/dL]	0.828
CE_LDL [mg/dL]	0.828
FC_LDL [mg/dL]	0.828
PL_LDL [mg/dL]	0.828
TG_LDL [mg/dL]	0.828
ApoB_LDL [mg/dL]	0.828
Mean_Part_Diam_LDL	-0.77
P_Num_IDL [nmol/L]	0.895
Chols_IDL [mg/dL]	0.895
CE_IDL [mg/dL]	0.895
FC_IDL [mg/dL]	0.895
PL_IDL [mg/dL]	0.895
TG_IDL [mg/dL]	0.854
ApoB_IDL [mg/dL]	0.895
P_Num_VLDL5 [nmol/L]	0.728
Chols_VLDL5 [mg/dL]	0.695
CE_VLDL5 [mg/dL]	0.695
FC_VLDL5 [mg/dL]	0.695
PL_VLDL5 [mg/dL]	0.695
TG_VLDL5 [mg/dL]	0.728
ApoB_VLDL5 [mg/dL]	0.728
P_Num_VLDL4 [nmol/L]	0.77
Chols_VLDL4 [mg/dL]	0.753
CE_VLDL4 [mg/dL]	0.753
FC_VLDL4 [mg/dL]	0.753
PL_VLDL4 [mg/dL]	0.753
TG_VLDL4 [mg/dL]	0.686
ApoB_VLDL4 [mg/dL]	0.753
P_Num_VLDL [nmol/L]	0.603
Chols_VLDL [mg/dL]	0.603
CE_VLDL [mg/dL]	0.603
FC_VLDL [mg/dL]	0.603
PL_VLDL [mg/dL]	0.603
ApoB_VLDL [mg/dL]	0.619
Mean_Part_Diam_CH [nm]	-0.612
Total_ApoB [mg/dL]%Total_ApoA1 [mg/dL]	0.77
Chols_LDL [mg/dL]%Chols_HDL [mg/dL]	0.619

### Comorbidities

#### Arterial hypertension

In hypertensive patients with SLE, SSc, and SD, only a single differential metabolite was identified compared with normotensive patients, respectively: threonine (decreased) in SLE, pyruvic acid (increased) in SSc, and carnitine (decreased) in SD. In hypertensive IIM patients in contrast, 11 substances were significantly reduced compared with normotensive individuals (Table 3). SLE patients showed 1 differently concentrated amino acid in hypertensive individuals, other metabolites from the 4 groups did not differ. For SSc and SD, there was only 1 significant difference in the other group. IIM patients differed in 11 lipids if arterial hypertension was present (supplemental Table 2).

**Table 3:** Metabolomic differences between hypertensive and non-hypertensive patients of the respective entities

metabolite	*p*-value (uncorr.)	*-notation (uncorr.)	*-notation (corr.)	fold change	cohen’s d
**SLE**					
Threonine	4.88e-02	*	n.s.	0.82	-0.75
**SSc**					
Carnitine	3.01e-02	*	n.s.	0.66	-1.27
**SD**					
Pyruvic acid	5.68e-03	**	n.s.	2.46	1.53
**IIM**					
Total_ApoA1 [mg/dL]	3.73e-02	*	n.s.	0.84	-1.74
Total_ApoA2 [mg/dL]	3.73e-02	*	n.s.	0.86	-1.84
Total_P_Num [nmol/L]	3.73e-02	*	n.s.	0.85	-1.92
Chols_HDL4 [mg/dL]	3.73e-02	*	n.s.	0.87	-2.03
CE_HDL4 [mg/dL]	3.73e-02	*	n.s.	0.87	-2.02
FC_HDL4 [mg/dL]	3.73e-02	*	n.s.	0.88	-2.04
PL_HDL4 [mg/dL]	3.73e-02	*	n.s.	0.87	-2.02
TG_HDL4 [mg/dL]	3.73e-02	*	n.s.	0.86	-1.99
P_Num_HDL [nmol/L]	3.73e-02	*	n.s.	0.85	-1.74
ApoA1_HDL [mg/dL]	3.73e-02	*	n.s.	0.84	-1.74
ApoA2_HDL [mg/dL]	3.73e-02	*	n.s.	0.86	-1.84

#### Diabetes mellitus

In patients with SLE, SSc, or SD who also had diabetes mellitus, numerous metabolites showed altered concentrations compared with non-diabetic patients (Table 4): SLE − 5 (all increased), SSc − 62 (5 increased, 57 decreased), SD − 20 (5 increased, 15 decreased). Because none of the IIM patients had diabetes mellitus, the corresponding analyses could not be conducted in this cohort (Table 4). If related to the 4 predefined groups, the following differences were found between diabetic and non-diabetic patients per entity: SLE – 1 carbohydrate, 4 other, 0 amino acids / lipids; SSc – 1 amino acid, 54 lipids, 1 carbohydrate, 6 other; SD – 4 amino acids, 1 lipid, 0 crabohydrates, 1 other; IIM – 4 amino acids, 15 lipids, 0 carbohydrates, 3 other (supplemental Table 2).

**Table 4:** Metabolomic differences between diabetic and non-diabetic individuals of the respective entities

metabolite	*p*-value (uncorr.)	*-notation (uncorr.)	*-notation (corr.)	fold change	cohen’s d
**SLE**					
1-Methylhistidine	1.39e-04	***	*	Inf	3.94
2-Hydroxyisobutyric acid	1.39e-04	***	*	Inf	3.94
Citric acid	3.15e-02	*	n.s.	1.35	1.52
Glucose	3.15e-02	*	n.s.	1.23	1.73
Cystine	4.49e-02	*	n.s.	2.21	1.74
**SSc**					
Mannose	1.29e-02	*	n.s.	1.67	2.35
3-Hydroxybutyric acid	1.78e-02	*	n.s.	5.47	3.34
Acetone	2.44e-02	*	n.s.	1.66	1.97
Glycine	2.44e-02	*	n.s.	0.62	-1.32
Leucine	2.44e-02	*	n.s.	1.31	2.07
Total_PL [mg/dL]	2.44e-02	*	n.s.	0.77	-1.31
P_Num_LDL5 [nmol/L]	2.44e-02	*	n.s.	0.60	-1.02
Chols_LDL5 [mg/dL]	2.44e-02	*	n.s.	0.60	-1.02
CE_LDL5 [mg/dL]	2.44e-02	*	n.s.	0.60	-1.02
FC_LDL5 [mg/dL]	2.44e-02	*	n.s.	0.60	-1.02
PL_LDL5 [mg/dL]	2.44e-02	*	n.s.	0.60	-1.02
TG_LDL5 [mg/dL]	2.44e-02	*	n.s.	0.60	-1.02
ApoB_LDL5 [mg/dL]	2.44e-02	*	n.s.	0.60	-1.02
P_Num_LDL4 [nmol/L]	2.44e-02	*	n.s.	0.62	-1.03
Chols_LDL4 [mg/dL]	2.44e-02	*	n.s.	0.62	-1.03
CE_LDL4 [mg/dL]	2.44e-02	*	n.s.	0.62	-1.03
FC_LDL4 [mg/dL]	2.44e-02	*	n.s.	0.62	-1.03
PL_LDL4 [mg/dL]	2.44e-02	*	n.s.	0.62	-1.03
TG_LDL4 [mg/dL]	2.44e-02	*	n.s.	0.62	-1.03
ApoB_LDL4 [mg/dL]	2.44e-02	*	n.s.	0.62	-1.03
P_Num_LDL3 [nmol/L]	2.44e-02	*	n.s.	0.64	-1.06
Chols_LDL3 [mg/dL]	2.44e-02	*	n.s.	0.64	-1.06
CE_LDL3 [mg/dL]	2.44e-02	*	n.s.	0.64	-1.06
FC_LDL3 [mg/dL]	2.44e-02	*	n.s.	0.64	-1.06
PL_LDL3 [mg/dL]	2.44e-02	*	n.s.	0.64	-1.06
TG_LDL3 [mg/dL]	2.44e-02	*	n.s.	0.64	-1.06
ApoB_LDL3 [mg/dL]	2.44e-02	*	n.s.	0.64	-1.06
P_Num_LDL2 [nmol/L]	2.44e-02	*	n.s.	0.66	-1.09
Chols_LDL2 [mg/dL]	2.44e-02	*	n.s.	0.66	-1.09
CE_LDL2 [mg/dL]	2.44e-02	*	n.s.	0.66	-1.09
FC_LDL2 [mg/dL]	2.44e-02	*	n.s.	0.66	-1.09
PL_LDL2 [mg/dL]	2.44e-02	*	n.s.	0.66	-1.09
TG_LDL2 [mg/dL]	2.44e-02	*	n.s.	0.66	-1.09
ApoB_LDL2 [mg/dL]	2.44e-02	*	n.s.	0.66	-1.09
P_Num_LDL [nmol/L]	2.44e-02	*	n.s.	0.63	-1.08
Chols_LDL [mg/dL]	2.44e-02	*	n.s.	0.64	-1.10
CE_LDL [mg/dL]	2.44e-02	*	n.s.	0.64	-1.10
FC_LDL [mg/dL]	2.44e-02	*	n.s.	0.64	-1.10
PL_LDL [mg/dL]	2.44e-02	*	n.s.	0.64	-1.10
TG_LDL [mg/dL]	2.44e-02	*	n.s.	0.64	-1.10
ApoB_LDL [mg/dL]	2.44e-02	*	n.s.	0.63	-1.08
Acetic acid	3.30e-02	*	n.s.	0.62	-1.07
Acetoacetic acid	3.30e-02	*	n.s.	2.29	2.12
Serine	3.30e-02	*	n.s.	1.24	1.52
Total_Chols [mg/dL]	3.30e-02	*	n.s.	0.74	-1.33
Total_CE [mg/dL]	3.30e-02	*	n.s.	0.74	-1.36
Total_FC [mg/dL]	3.30e-02	*	n.s.	0.74	-1.23
P_Num_LDL6 [nmol/L]	3.30e-02	*	n.s.	0.61	-1.04
Chols_LDL6 [mg/dL]	3.30e-02	*	n.s.	0.60	-1.04
CE_LDL6 [mg/dL]	3.30e-02	*	n.s.	0.60	-1.04
FC_LDL6 [mg/dL]	3.30e-02	*	n.s.	0.60	-1.05
PL_LDL6 [mg/dL]	3.30e-02	*	n.s.	0.61	-1.04
TG_LDL6 [mg/dL]	3.30e-02	*	n.s.	0.61	-1.04
ApoB_LDL6 [mg/dL]	3.30e-02	*	n.s.	0.61	-1.04
P_Num_LDL1 [nmol/L]	3.30e-02	*	n.s.	0.67	-1.16
Chols_LDL1 [mg/dL]	3.30e-02	*	n.s.	0.67	-1.17
CE_LDL1 [mg/dL]	3.30e-02	*	n.s.	0.67	-1.17
FC_LDL1 [mg/dL]	3.30e-02	*	n.s.	0.67	-1.17
PL_LDL1 [mg/dL]	3.30e-02	*	n.s.	0.67	-1.19
TG_LDL1 [mg/dL]	3.30e-02	*	n.s.	0.67	-1.19
ApoB_LDL1 [mg/dL]	3.30e-02	*	n.s.	0.67	-1.18
Mean_Part_Diam_VLDL4 [nm]	3.30e-02	*	n.s.	1.00	1.91
**SD**					
Isopropanol	9.12e-03	**	n.s.	8.57	2.84
Methionine	1.40e-02	*	n.s.	Inf	2.65
Phenylalanine	3.21e-02	*	n.s.	2.38	3.12
Serine	3.21e-02	*	n.s.	1.37	1.96
Tyrosine	3.21e-02	*	n.s.	1.68	2.72
P_Num_VLDL3 [nmol/L]	4.72e-02	*	n.s.	0.34	-1.36
Chols_VLDL3 [mg/dL]	4.72e-02	*	n.s.	0.34	-1.36
CE_VLDL3 [mg/dL]	4.72e-02	*	n.s.	0.34	-1.36
FC_VLDL3 [mg/dL]	4.72e-02	*	n.s.	0.34	-1.36
PL_VLDL3 [mg/dL]	4.72e-02	*	n.s.	0.34	-1.36
TG_VLDL3 [mg/dL]	4.72e-02	*	n.s.	0.34	-1.36
ApoB_VLDL3 [mg/dL]	4.72e-02	*	n.s.	0.34	-1.36
P_Num_VLDL2 [nmol/L]	4.72e-02	*	n.s.	0.30	-1.25
Chols_VLDL2 [mg/dL]	4.72e-02	*	n.s.	0.30	-1.24
CE_VLDL2 [mg/dL]	4.72e-02	*	n.s.	0.29	-1.24
FC_VLDL2 [mg/dL]	4.72e-02	*	n.s.	0.30	-1.24
PL_VLDL2 [mg/dL]	4.72e-02	*	n.s.	0.30	-1.24
TG_VLDL2 [mg/dL]	4.72e-02	*	n.s.	0.30	-1.24
ApoB_VLDL2 [mg/dL]	4.72e-02	*	n.s.	0.30	-1.25
TG_VLDL [mg/dL]	4.72e-02	*	n.s.	0.34	-1.28

#### Stress

Stress or distress was associated with a significant decrease in the concentrations of 21 metabolites in SLE patients (1 amino acid, 16 lipids, 4 other). In contrast, 13 metabolites were significantly elevated in SSc when inadequate stress was reported (11 lipids, 2 other). In SD patients, only methanol was found in higher concentrations in the stress cohort. IIM was characterized by elevated concentrations of tyrosine, provided that increased stress was reported (supplemental Table 1).

#### Smoking

The following significant differences were found between smokers and non-smokers with regard to the respective entities: SLE – 5 higher concentration metabolites, 2 lower concentration metabolites (3 amino acids, 1 lipid, 1 carbohydrate, 2 other); SSc – one elevated metabolite (other); SD – 9 elevated metabolites, 4 reduced metabolites (2 amino acids, 7 lipids, 4 other); IIM – 4 elevated metabolites (1 amino acid, 1 carbohydrate, 2 other) (supplemental Table 1).

### Laboratory findings

#### Total cholesterol LDL, and HDL cholesterol

In patients with SLE, more than 70 metabolites demonstrated a positive correlation with total cholesterol (70 lipids, 2 other); comparable associations were observed in cases of SSc (1 amino acid, 74 lipids, 2 other), SD (88 lipids, 1 other), and IIM (4 amino acids, 86 lipids, 4 other). A limited number of metabolites demonstrated negative correlations. Similar results were observed in relation to LDL cholesterol. Each entity was associated with a minimum of 60 correlations, the majority of which were positive (SLE – 104 lipids, 1 other, SSc – 62 lipids, 1 other, SD – 79 lipids, 1 carbohydrate, 1 other; IIM – 1 amino acid, 83 lipids, 1 carbohydrate, 2 other). Finally, HDL was also associated with large numbers of metabolites in each individual type of ACTD, the majority of all associations were also identified as positive correlations, respectively (SLE – 52 lipids, 1 other, SSc – 1 amino acid, 76 lipids, 1 carbohydrate, 2 other, SD – 33 lipids, 5 other, IIM – 2 amino acids, 55 lipids, 6 other). Comprehensive numerical data are provided in the supplemental Table 1.

#### Urine albumin-creatinine ratio (UACR)

SLE and IIM patients showed no associations or correlations between distinct metabolites and UACR. In SSc, two positive correlations were found (dimethylglycine CC 0.697; myo-inositol CC 0.752). SD patients showed one positive correlation: Mean_Part_Diam_HDL4 [nm] CC 0.61.

## Discussion

It is challenging to offer a comprehensive interpretation of the extensive data sets presented in this study. The authors have determined that the following categories are the most appropriate ones to be discussed as they correspond to conventional cardiovascular risk factors.

### Body mass index

As summarized in detail by Regan and Shah 2020 [18], branched-chain amino acids (BCAA - valine, leucine, isoleucine) are particularly suitable for differentiating between overweight and normal-weight individuals. A corresponding study by Newgard et al. (2009) [19] led to the identification of this specific amino acid subgroup in overweight individuals. Our analysis revealed only two positive correlations between BMI and the amino acids valine and isoleucine in the SSc cohort. No abnormalities were found in the remaining cohorts (SLE, SD, and IIM) with regard to BCAA. In 2013, Weir et al. [20] reported associations between BMI and cholesterol esters (CE), triacylglycerol (TAG), and ceramides (CER). Comparable findings could currently only be confirmed in one cohort, namely patients with SSc. A total of 13 lipid components correlated positively with BMI, including CE, TAG, and CER. In summary, the findings regarding BCAAs in patients with ACTD appear to be of limited relevance, and abnormalities in lipid metabolism documented in the literature cannot be consistently confirmed. The observed absence of any correlations between BMI and distinct metabolites in SLE was unexpected, especially given the known significantly increased cardiovascular risk in SLE. A surprising observation was 13 significant correlations between BMI and lipid metabolites in individuals with SSc, only SD was characterized by 1 significant correlation. The other two entities did not show any abnormalities in this regard. All lipids were variants of very low-density lipoproteins. From this special population, it may be possible to select distinct candidates in order to check a possible suitability in the CVR assessment at SSc.

### Framingham score

In 2023, a comprehensive analysis of over 3,000 participants in the Framingham Heart Study cohort was published. The objective of the study was to identify associations between metabolomic characteristics and the AHA Cardiovascular Health Score (AHA CHS), as well as the endpoints of incident heart failure and incident atrial fibrillation [21]. Unlike the Framingham score, the AHA CHS takes a larger number of variables into account with the aim of optimizing cardiovascular risk prediction. The metabolites associated with three specific cardiometabolic components of the CVH score (ideal BMI, blood pressure, and fasting blood sugar) were of particular importance for amino acid metabolism, especially in the glucose-alanine cycle, alanine metabolism, and lactose breakdown. The current analysis revealed no abnormalities in alanine metabolism in any of the entities examined. SLE patients exhibited exclusively positive correlations of various lipoproteins, while in SSc, cystine demonstrated a positive correlation with the Framingham score, and in SD, pyruvic acid and taurine also exhibited positive correlations. Finally, a multitude of predominantly positive correlations were identified between various lipoproteins and the score in IIM. The observed differences in lipoprotein profiles between IIM and SLE patients present an unexpected finding, particularly given that cardiovascular disease risk is similarly heightened in both groups. In 2023, Bello et al. [22] conducted a comprehensive meta-analysis encompassing more than 40 studies involving SLE patients, concluding that the relative risks for stroke, myocardial infarction, and arterial hypertension were elevated by factors ranging from 2.5 to 2.9. Similarly, Xiong et al. [23] identified an approximate 2.4-fold increase in these risks among individuals with IIM. Studies conducted in 2021 show that IIM patients have a noticeably different lipoprotein profile compared to healthy people [24].

### Arterial hypertension

Since the mid-2000s, an increasing number of publications on metabolomics in arterial hypertension have been written each year compared to the previous year. Firstly, it is important to note that many amino acids demonstrate dysregulation, with branched-chain amino acids (BCAAs) such as leucine often elevated, and valine and isoleucine frequently reduced, in hypertensive individuals [25]. Furthermore, certain aromatic amino acids (e.g. phenylalanine) have also been found to be elevated, while tryptophan and tyrosine have frequently been observed to be reduced in hypertension [26]. Low glycine levels have been suggested to contribute to elevated blood pressure [27]. Glycine deficiency is believed to impair endothelial function and nitric oxide bioavailability [28]. Lipid-related metabolite changes are also common. For instance, acylcarnitines (markers of fatty acid oxidation) accumulate in hypertension, as do certain fatty acids, such as oleic acid and ceramide [28]. Conversely, beneficial short-chain fatty acids (SCFAs) derived from gut microbiota appear to be reduced in hypertensive individuals [29]. Given the complexity of the data, which we can only roughly outline here, our results are quite unexpected. In SLE - where cardiovascular risk is similarly high as in IIM [22, 23] - we anticipated that more metabolomic indicators would be linked to arterial hypertension. However, only one metabolite was found to differ between SLE patients with and without hypertension, and this result was mirrored in both SSc and SD. In IIM patients, 11 metabolites differed between normotensive and hypertensive individuals; all substances were lipoproteins. However, when interpreting these findings, it is important to note that typical, albeit non-specific, metabolic aberrations in arterial hypertension are not consistently reproducible in the context of ACTD. All groups showed an increased risk of cardiovascular disease, which is especially important to note. This study revealed only minor differences in metabolic profiles between hypertensive and normotensive patients with ACTD, marking a significant finding. Abnormal lipid profiles appeared solely in the IIM group and were absent from the other groups.

### Diabetes mellitus

In comparison to arterial hypertension, metabolomic research related to diabetes mellitus has been conducted at an even higher frequency over the past two decades. Given the metabolic nature of the disease, this is more than plausible. The following objectives have already been addressed: identification of early diagnostic markers (branched-chain amino acids, lysophosphatidylcholines [30]), pathogenesis of the disease (acylcarnitines, ceramides [31]), drug response (lactate, bile acids [32]), and monitoring of complications (kidneys, nervous system - sorbitol, uric acid [33]). Our study revealed multiple differences in metabolite concentrations between non-diabetic and diabetic patients with ACTD. In contrast to the variables already discussed (e.g., Framingham score, arterial hypertension), however, no differences in the metabolome could be identified in IIM patients with regard to diabetes mellitus, since none of the IIM individuals was diagnosed as diabetic. The highest number of differences was found in SSc (*n* = 62), followed by SD (*n* = 20) and SLE (*n* = 5). In the SSC cohort, differences were found in the following substances or substance groups: mannose, 3-hydroxybutyric acid, acetone, glycine, leucine, and various lipoproteins. The increase in cardiovascular risk in SSc was investigated in a meta-analysis in 2024 [34]. For various endpoints (stroke, myocardial infarction, peripheral artery disease), hazard ratios ranging from 1.64 (stroke) to 5.23 (peripheral artery disease) were observed. Cardiovascular risk in SSc appears at least on par with the risks seen in SLE and IIM. It’s uncertain whether common dyslipidemia in SSc indicates a unique type of endothelial damage. Nonetheless, when considering our study’s principal aim, the lipid profile of SSc patients could reveal promising candidate molecules.

### Regular glucocorticoid therapy and daily prednisolone dose

The prevalence rates of regular GC use were as follows: SLE − 81%, SSc − 66%, SD − 75%, and IIM − 55%. Analysis of metabolite profiles comparing individuals with and without regular GC intake revealed significant differences in lipid metabolism for SLE and SSc. Interpretation of metabolomic deviations during established GC therapy remains challenging due to the well-documented substantial metabolic effects of these agents on protein, carbohydrate, and lipid metabolism, which contribute to the complexity of long-term drug administration [35]. To eliminate these effects on the metabolome, it would theoretically require a GC-free study design; however, such an approach raises significant ethical concerns. There is considerable uncertainty regarding whether the observed changes in the lipid metabolome in SLE and SSc are primarily associated with GC use, given the lack of correlation with daily dosage in SD (where the GC usage rate is 75%). Nevertheless, certain candidates within the lipid group may be selected to evaluate their suitability for CVR assessment in the future.

### Dyslipidemia

It is important to recognize that dyslipidemias, regardless of the specific condition, already affect the lipid metabolome itself. For instance, while 104 correlations involving LDL cholesterol were found in SLE and only 62 in SSc, we believe that drawing any conclusions - even tentative ones - would be questionable. All that remains, then, is to make the data available to the public.

### Urine albumine-to-creatinine ratio (UACR)

Only three metabolites correlated with UACR: dimethylglycine and myo-inositol in SSc, and Mean_Part_Diam_HDL4 in SD. Although SLE in particular is frequently associated with kidney involvement, which also has great prognostic significance in the disease, no associations were found. In 2023, myo-inositol has been identified as a urinary biomarker of diabetic nephropathy progression [36]. In contrast, no association between dimethylglycine and proteinuria has been reported so far.

Drawing a definitive conclusion remains challenging. Notably, a significant number of metabolites were found to be abnormally concentrated in individuals with ACTD, particularly when certain cardiovascular risk factors are present. It is noteworthy that, within this specific patient group, metabolomic profiles previously reported for cardiovascular risk individuals without ACTD are only occasionally observed (such as BMI category, isoleucine levels in SSc). This observation likely corresponds to the central finding of the entire study: relative to the general population, specific cardiovascular risk factors are associated with distinct metabolomic profiles in ACTD patients. All entities investigated exhibit a highly complex and largely unresolved pathogenesis. It is reasonable to consider that the diverse disturbances in immune homeostasis observed across all forms may correspond to similarly varied alterations in the metabolomic profile. The possible diagnostic implications are equally difficult to identify, not least because of the low prevalence of the diseases. The data at least suggest that potentially diverse lipid components should be considered as the most suitable candidates in follow-up studies, which should in any case be longitudinal and would also have to take into account cardiovascular end-organ damage as well as secondary and concomitant diseases.

## Limitations

The most significant limitation is undoubtedly the small size of all the analyzed groups. This circumstance is a consequence of the low prevalence of the diseases in general, especially in the case of SSc and IIM. The relatively small sample size was the most important reason why multivariate analyses were not conducted. Such analyses would, of course, have been helpful. Another limitation is the lack of follow-up data. This complicates the assessment of potential changes in the metabolome in the case of cardiovascular events (e.g., myocardial infarction), for instance. Information regarding cardiovascular end-organ damage or secondary and comorbid conditions was also not taken into account. An additional limitation is the predominant daily use of GC. The numerous metabolic effects of the drugs are likely to modify the metabolome in the long term. It is important to reiterate - as highlighted by the study’s title - that the current project is exploratory in nature. Currently, specific substrates cannot be regarded as diagnostically valuable, and, of course, no candidates can yet be classified as biomarkers. The study’s design does not permit such conclusions.

## Supplementary Information

Below is the link to the electronic supplementary material.

Supplementary Material 1 Numbers of all statistically significant metabolomic differences between the categorical data (gender, regular glucocorticoid therapy, stress, smoking) and all significant correlations between metabolites and non-categorical data (BMI, daily prednisolone dose, total cholesterol, LDL and HDL cholesterol)

Supplementary Material 2 Numbers of all statistically significant metabolomic differences between the categorical data arterial hypertension and diabetes mellitus and all significant correlations between metabolites and the Framingham score

## Data Availability

All data will be provided by d.patschan@gmail.com upon reasonable request.
