# Supplementary material for: Metabolomics and cardiovascular risk factors in autoimmune-mediated connective tissue diseases – an exploratory, hypothesis-generating study
Source: BMC Rheumatol. 2026 Jul 1;10:54. doi: 10.1186/s41927-026-00670-8 (PMC13326006; doi:10.1186/s41927-026-00670-8)
Supplement: Supplementary file 1 — Supplementary Material 1 Numbers of all statistically significant metabolomic differences between the categorical data (gender, regular glucocorticoid therapy, stress, smoking) and all significant correlations between metabolites and non-categorical data (BMI, daily prednisolone dose, total cholesterol, LDL and HDL cholesterol) [file 41927_2026_670_MOESM1_ESM.docx]

|  | amino acid metabolism | lipid metabolism | carbohydrate metabolism | other |
| --- | --- | --- | --- | --- |
| **age** | | | | |
| **SLE** | 0 | 16 | 0 | 0 |
| **SSc** | 2 | 0 | 0 | 1 |
| **SD** | 0 | 0 | 0 | 1 |
| **IIM** | 3 | 11 | 0 | 3 |
| **gender** | | | | |
| **SLE** | 2 | 0 | 0 | 3 |
| **SSc** | 6 | 27 | 0 | 5 |
| **SD** | 0 | 0 | 0 | 0 |
| **IIM** | 1 | 0 | 0 | 1 |
| **BMI** | | | | |
| **SLE** | 0 | 0 | 0 | 0 |
| **SSc** | 2 | 13 | 2 | 1 |
| **SD** | 2 | 1 | 1 | 1 |
| **IIM** | 2 | 0 | 0 | 3 |
| **regular glucocorticoid therapy** | | | | |
| **SLE** | 0 | 7 | 0 | 4 |
| **SSc** | 0 | 7 | 0 | 1 |
| **SD** | 1 | 3 | 0 | 2 |
| **IIM** | 1 | 1 | 0 | 1 |
| **daily prednisolone dose** | | | | |
| **SLE** | 0 | 22 | 0 | 0 |
| **SSc** | 1 | 23 | 0 | 2 |
| **SD** | 0 | 0 | 0 | 1 |
| **IIM** | 0 | 0 | 0 | 0 |
| **stress** | | | | |
| **SLE** | 1 | 16 | 0 | 4 |
| **SSc** | 0 | 11 | 0 | 2 |
| **SD** | 0 | 0 | 0 | 1 |
| **IIM** | 1 | 0 | 0 | 0 |
| **smoking** | | | | |
| **SLE** | 3 | 1 | 1 | 2 |
| **SSc** | 0 | 0 | 0 | 1 |
| **SD** | 2 | 7 | 0 | 4 |
| **IIM** | 1 | 0 | 1 | 2 |
| **total cholesterol** | | | | |
| **SLE** | 0 | 70 | 0 | 2 |
| **SSc** | 1 | 74 | 0 | 2 |
| **SD** | 0 | 88 | 0 | 1 |
| **IIM** | 4 | 86 | 0 | 4 |
| **LDL cholesterol** | | | | |
| **SLE** | 0 | 104 | 0 | 1 |
| **SSc** | 0 | 62 | 0 | 1 |
| **SD** | 0 | 79 | 0 | 0 |
| **IIM** | 1 | 83 | 1 | 2 |
| **HDL cholesterol** | | | | |
| **SLE** | 0 | 52 | 0 | 1 |
| **SSc** | 1 | 76 | 1 | 2 |
| **SD** | 0 | 33 | 0 | 5 |
| **IIM** | 2 | 55 | 0 | 6 |
