# Supplementary material for: Metabolomics and cardiovascular risk factors in autoimmune-mediated connective tissue diseases – an exploratory, hypothesis-generating study
Source: BMC Rheumatol. 2026 Jul 1;10:54. doi: 10.1186/s41927-026-00670-8 (PMC13326006; doi:10.1186/s41927-026-00670-8)
Supplement: Supplementary file 2 — Supplementary Material 2 Numbers of all statistically significant metabolomic differences between the categorical data arterial hypertension and diabetes mellitus and all significant correlations between metabolites and the Framingham score [file 41927_2026_670_MOESM2_ESM.docx]

|  | amino acid metabolism | lipid metabolism | carbohydrate metabolism | other |
| --- | --- | --- | --- | --- |
| **Framingham score** | | | | |
| **SLE** | 0 | 11 | 0 | 0 |
| **SSc** | 0 | 0 | 0 | 1 |
| **SD** | 0 | 0 | 0 | 2 |
| **IIM** | 4 | 86 | 0 | 3 |
| **arterial hypertension** | | | | |
| **SLE** | 1 | 0 | 0 | 0 |
| **SSc** | 0 | 0 | 0 | 1 |
| **SD** | 0 | 0 | 0 | 1 |
| **IIM** | 0 | 11 | 0 | 0 |
| **diabetes mellitus** | | | | |
| **SLE** | 0 | 0 | 1 | 4 |
| **SSc** | 1 | 54 | 1 | 6 |
| **SD** | 4 | 15 | 0 | 1 |
| **IIM** | not applicable | | | |
